# Community-Dwelling People Living With Dementia and Their Family Caregivers Experience Enhanced Relationships and Feelings of Well-Being Following Therapeutic Group Singing: A Qualitative Thematic Analysis

**DOI:** 10.3389/fpsyg.2018.01332

**Published:** 2018-07-30

**Authors:** Imogen N. Clark, Jeanette D. Tamplin, Felicity A. Baker

**Affiliations:** Faculty of Fine Arts and Music, The University of Melbourne, Melbourne, VIC, Australia

**Keywords:** group singing, people living with dementia, family caregivers, qualitative thematic analysis, community-dwelling

## Abstract

The progression of dementia can severely compromise interpersonal connection and relationship quality between people living with dementia (PwD) and their family caregivers (FCG), leading to social isolation and poor quality of life for both. Therapeutic group singing (TGS) is a socially engaging, stimulating, and supportive pursuit that community-dwelling PwD and their FCG can participate in together. This study aimed to build on the findings from previous research by undertaking a thematic analysis of interviews with nine PwD (five women, four men; mean age = 79.1 years) and nine FCG (five women, four men; mean age = 75.7 years). The interviews explored participants’ perspectives and experiences of a 20-week TGS intervention, underpinned by Kitwood’s model of person-centered care. Inductive thematic analysis resulted in the emergence of five themes which described how TGS for PwD and their FCG: (1) included supportive therapeutic facilitation and design features; (2) made group singing more accessible; (3) fostered new empathic friendships; (4) enhanced relationships between PwD and FCG; and (5) led to personal feelings of wellbeing for both PwD and FCG. Affinity with others who had similar life experiences and challenges created a sense of mutual understanding and camaraderie, which made group singing accessible without fear of judgment and social stigmas. For some PwD/FCG dyads, TGS meant they could continue a lifelong passion for singing together, while others enjoyed participating in singing together for the first time. Both PwD and FCG participants described personal feelings of acceptance, improved social confidence, mood, and purpose. Further, participants valued mental stimulation from TGS such as learning new skills and memory support. A model explaining relationships between themes suggests that TGS with person-centered facilitation features for PwD/FCG dyads led to affinity among group members with ripple effects, which enhanced accessibility to group singing, the formation of empathic friendships, PwD/FCG relationship quality, and personal wellbeing for both PwD and FCG. Psychoemotional, social and cognitive benefits from TGS described by participants in this study are known to promote self-identity, healthy relationships, and quality of life. This research highlights a need for improved availability of TGS for community-dwelling PwD/FCG dyads.

## Introduction

Dementia currently compromises the health and quality of life for ∼47 million people and their families worldwide, and this figure is expected to reach 131 million by 2050 (Prince et al., 2016). A high proportion of people living with dementia (PwD) reside in the community and considerable societal global costs, estimated to be around $330.8 billion US, are attributed to informal care provided by family caregivers (FCG) (Prince et al., 2015). Estimates report that numbers of informal FCG, usually a spouse/partner and less frequently a son/daughter/other relation or friend, are around 670,000 in the United Kingdom (Lewis et al., 2014), 3.6 million in the United States (Friedman et al., 2015), and 200,000 in Australia (Ross and Beattie, 2015). The familiarity of a long-term residence supports optimal independence for PwD and also reduces costs to families and to society (Greenblat, 2012). Therefore, a strategic priority recognized by the World Health Organization aims to support community-dwelling PwD/FCG dyads to live together for as long as possible in the family home of their choice (Greenblat, 2012). However, FCG often provide unpaid care for several years from early diagnosis (or before) and accept escalating workloads as personal care and supervision needs increase (Greenblat, 2012). As such, FCG are prone to high burden of care along with accompanying physical and mental health conditions, which in turn increase the likelihood for negative symptoms in PwD and placement in residential care (Prince et al., 2016).

Community psychosocial services that aim to support emotional coping for FCG, independence for PwD, and social engagement, health, and emotional wellbeing for both are known to delay admission to residential care for PwD, particularly when commenced early in the disease trajectory (Greenblat, 2012; Prince et al., 2015; Farina et al., 2017; Frankish and Horton, 2017). For PwD, symptoms such as memory loss and aphasia, may lead to reduced confidence in social situations, which when coupled with social stigmas, can result in withdrawal and isolation from previously meaningful activities (Burgener et al., 2015a,b). To combat these experiences, Kitwood’s (1997) landmark model recognizes person-centered care with respectful trusting relationships and social connectedness as essential if “personhood” is to be maintained by PwD in the face of cognitive decline.

Kitwood’s person-centered care model seeks to foster autonomy, independence and social skill in PwD, acknowledging that life can be well lived regardless of deficits in memory, reasoning, communication, and capacity to care for self (Kitwood, 1997). Within this framework, personhood is regarded as a status that is bestowed on one person (the PwD) by another (the caregiver). Rather than viewing cognitive impairment and compounding factors such as agitation, anxiety, apathy and depression as negative consequences of dementia, caregivers and recipients are co-contributors in an intersubjective relationship fostering relative wellbeing (Kitwood and Bredin, 1992). Twelve concepts guiding Kitwood’s person-centered approach are recognized for support relative wellbeing in PwD: (1) Recognition of each person as unique; (2) Negotiation ensuring personal preferences are considered for all aspects of daily life; (3) Collaboration between care recipient and giver in decision making; (4) Play involving preferred activities that enable self-expression; (5) Giving, denoting recognition of acts of kindness from PwD toward caregivers and others; (6) Timalation involving interactions using aesthetic sensual experiences; (7) Celebration of everyday achievements; (8) Relaxation facilitated by modification to environmental stimuli; (9) Validation and acceptance of the person’s reality; (10) Holding a safe psychological space for expression; (11) Creation and self-expression through art forms such as music; and (12) Facilitation of tasks that are challenging (Kitwood, 1997). Fazio et al.’s (2018) recent review of articles examining person-centered care acknowledges that these concepts remain extant in current dementia care practices and research.

While FCG are often acutely aware of the need to sustain personhood in their loved one living with dementia (Wadham et al., 2016), they too are subject to experiences of social isolation resulting from the responsibility of day to day care and their loved one’s unpredictable behavior during social situations (Greenblat, 2012; Nay et al., 2015). To reduce this risk of social isolation for both PwD and FCG, accessibility to dyadic interventions that place an emphasis on the PwD/FCG partnership and shared identity rather than focussing solely on either the PwD or FCG as individuals are encouraged (Nay et al., 2015; Spector et al., 2016; Wadham et al., 2016). Further, strengths-based rather than deficit orientated psychosocial services and interventions are particularly recommended to promote healthy relationship quality between PwD and FCG (Kitwood and Bredin, 1992; Hellström et al., 2005; Merrick et al., 2016; Wadham et al., 2016).

Therapeutic group singing (TGS) is a low-cost intervention that may support personhood in PwD, offer meaningful shared experiences for PwD and FCG, and improve health and wellbeing for both (Clark and Harding, 2012; Yates et al., 2016). Recent evidence from a systematic review with 18 studies suggests that stimulation associated with singing supports cognitive function among older people with various age-related health conditions including dementia (Yates et al., 2016). Implicit musical memory, that is the ability to sing and play an instrument, is retained into the late stages of dementia lending weight to the notion that active music participation supports memory function (Baird and Samson, 2009). Further systematic reviews investigating the effects of therapeutic singing interventions for PwD facilitated by credentialed music therapists in residential care contexts demonstrated improved mood and reductions in behavioral disturbances and depressive symptoms (McDermott et al., 2013; van der Steen et al., 2017). Individualized singing interventions implemented by FCG in the family home have also led to reduced physical signs of depression and improved mood, orientation, and episodic memory in PwD, and improved short-term memory, working memory and wellbeing in FCG compared with usual care (Sarkamo et al., 2013). Beyond the amelioration of psychological and behavioral symptoms, TGS is also a potent stimulus of interpersonal and intrapersonal social connection for PwD and their FCG (McDermott et al., 2014).

With the increasing acknowledgment of benefits from singing for PwD, there is a growing demand for singing groups or choirs for community-dwelling PwD and their FCG (for example, “Singing for the Brain” in the United Kingdom, which currently offers over 100 singing groups and has long waiting lists) (Osman et al., 2016). Unadkat et al. (2017) examined this phenomenon using grounded theory to analyze interview data from 17 PwD/FCG spousal dyads who had attended various choirs across the United Kingdom. They proposed that group singing is an accessible and joyful activity for both PwD and FCG, which when combined with effective facilitation leads to feelings of social belonging and connection, and ultimately individual benefits for both PwD and FCG, and as a couple (Unadkat et al., 2017). Other recent research supports this notion in suggesting that community singing groups attended by PwD and their FCG support experiences of wellbeing, social inclusiveness and connectedness, improved relationship quality with each other and others, opportunities for learning, and acceptance and coping with dementia (Camic et al., 2013; Osman et al., 2016; Unadkat et al., 2017). Davidson and Almeida (2014) further demonstrate improved mood for both PwD and FCG participants and relaxation levels, lucidity and focus in PwD following 6-weekly group singing sessions. These findings suggest that group singing is a creative normalizing activity offering health and wellbeing benefits for PwD and FCG as a dyad and as individuals.

Previous research has provided initial evidence of benefits for PwD and FCG from community singing groups (Camic et al., 2013; Davidson and Almeida, 2014; Osman et al., 2016; Unadkat et al., 2017), and therapeutic singing groups in residential care (McDermott et al., 2013; van der Steen et al., 2017). However, to the best of our knowledge there has been no research describing therapeutic community-based group singing interventions for PwD and their FCG facilitated by credentialed music therapists. The current project, funded by the National Health and Medical Research Council and Australian Research Council (APP1106603), therefore extends previous research with an exploration of community-dwelling PwD and FCGs’ experiences of TGS underpinned by Kitwood’s person-centered care model. Qualitative interviews sought to investigate the feasibility of our TGS intervention from the perspectives of our participants.

## Materials and Methods

### Research Design

The current paper reports thematic analysis of qualitative interviews conducted with participants following a pre–post feasibility trial.

### Participants

We recruited 12 PwD/FCG dyads to examine the feasibility of our TGS intervention. Allowing for attrition, this sample size was considered sufficient for capturing differing perceptions of the TGS experience (Creswell and Poth, 2018). PwD and FCG registered their interest in the project following attendance at information sessions and/or after receiving an information flyer from community organizations offering dementia support services. PwD/FCG dyads had to be living together in their own home in the community to be eligible for this study. PwD were eligible for the study if they had a clinical diagnosis of mild to moderate dementia with a Mini Mental State Exam score between 10 and 26 (Folstein et al., 1975). FCG were eligible for the study if they were the primary care giver for the PwD. Both PwD and FCG participants needed to have functional hearing with or without hearing aids and speak English. Approval for this study was obtained from the relevant health service human research ethics committee (approval number HREC/15/Austin/445). Written informed consent was obtained from all participants (FCG and PwD). In cases where researchers were uncertain of a PwD’s capacity to provide written informed consent, a person responsible who was not a participant in the study (not their FCG) was also asked to complete written informed consent on behalf of the PwD.

### Singing and Music Interventions

The intervention consisted of 20 TGS sessions that PwD and FCG participants attended together. Owing to a staggered recruitment process, participants commenced and completed their 20 sessions on differing dates over a 12-month period. Participants who had completed their final data collection assessment were encouraged to keep attending the TGS sessions if they wished. TGS sessions were held in a spacious room at a large public health facility. The number of participants in the group was limited to 15 PwD/FCG dyads (30 participants in total).

Each TGS session, held over ∼120 min, included introductions and information updates (5–10 min), vocal warm ups and exercises (15–20 min), singing familiar participant requested songs (30–45 min), learning new songs and singing skills introduced by the researchers (20–30 min), and socialization over afternoon tea (30 min). Most sessions were facilitated by two registered music therapists (also the researchers – authors 1 and 2), although occasional sessions were facilitated by only one therapist. Registered music therapists (RMTs) in Australia have completed an accredited tertiary course and maintain ongoing professional development (Australian Music Therapy Association, 2017). Additional support was provided by volunteers and student music therapists over the 12-month intervention period. A power-point presentation with an index of ∼75 participant nominated songs and accompanying lyrics for each song was used to support singing. The RMTs used guitars, keyboard, and banjo to accompany singing. Occasionally, participants were also invited to play percussion instruments. To support participant involvement and ownership of the group, PWD and FCG were encouraged to contribute to the running of the group with tasks such as setting up afternoon tea, marking attendance on a list, giving out name tags, and taking tea and coffee orders.

Twelve concepts of person-centered care developed by Kitwood (1997) were used to guide the facilitation of TGS sessions in an effort to maximize positive experiences for both PwD and FCG, and to mitigate any potential negative feelings and behaviors for PwD (such as apathy, agitation, and anxiety). **Table 1** describes how each of these concepts were integrated into TGS sessions.

**Table 1:** Facilitation of therapeutic group singing sessions based on Kitwood’s concepts of personhood.

Concept	Therapeutic group singing facilitation practice
Recognition	Recognition of individual preferences such as music tastes, seating options, and level of participation (overt or covert). Name tags worn by all participants and facilitators to compensate for memory loss.
Negotiation	All members of the group were encouraged to contribute to group decision making processes. This meant that some members required more support than others to ensure the musical and non-musical preferences of each person were heard and incorporated into sessions.
Collaboration	All major decisions involved collaboration between the participants and facilitators. For example, decisions regarding performance invitations, choice of venue, length of sessions, and preferred days of the week to meet.
Play	Encouragement of musical and non-musical playful self-expression including humor and creative movement.
Giving	Acknowledgment and acceptance of kind and generous contributions from participants.
Timalation	Modulating elements in the music (such as rhythm, harmony, tempo, lyrics) to meet the aesthetic needs of the group, and individuals within the group as required.
Celebration	Joyous, overt, and frequent celebrations of musical achievements by individuals including applause for solo singing, instrument playing or dancing. Celebration of non-musical events such as birthdays and anniversaries. Group celebrations following performances.
Relaxation	Considering of session pacing, including provision of quieter, more reflective songs, in addition to upbeat active music. Provision of space for individuals to seek solitude or to just listen to the music passively if desired.
Validation	Validating and accepting the experienced reality for each individual regardless of actual events.
Holding	Therapeutic use of music coupled with therapeutic listening and conversational skills to meet the needs of the group, provide a safe psychological space for individual self-expression, and promote peer support. Providing individual attention for a particular participant by one facilitator if needed.
Creation	Encouragement of creative contributions such as individual singing, song parody, harmonies (part singing), movement/dance, and instrumental contributions.
Facilitation	Encouragement to participate in challenging and cognitively stimulating active singing and music-making opportunities such as part singing, rounds, learning new songs, songwriting, and instrumental contributions.


In addition to the regular TGS sessions, we provided participants with music-based resources for use at home between sessions. These resources included recordings of songs used in TGS sessions and personalized play lists.

### Interviews and Thematic Analysis

PwD/FCG dyads were invited to participate in semi-structured interviews together. Interviews, facilitated by Authors 1 and 2, were audio-recorded and transcribed by Author 1 for analysis. The following questions were used to guide interviews: (1) Why did you agree to participate in this project? (2) What, if any, benefits were you expecting? (3) Did the singing group meet your expectations? Why or why not? (4) How would you describe your experience of the group? (5) How did being part of a singing group make you feel? (6) Was there anything additional that you would have liked the group sessions to offer? (7) How could the music therapy program be improved to suit your individual needs? (8) What, if anything, did you learn from participating in this project? (9) Would you encourage other people with dementia and their caregivers to participate in singing groups? Why or why not?

Inductive thematic analysis was performed using guidelines outlined by Braun and Clarke (2006). A systematic analysis of interview data involved the following steps: (1) Author 1 transcribed recorded interviews verbatim in word documents and imported these into MAXQDA12 for analysis (VERBI Software, 2017). (2) Author 1 read through the transcripts several times to gain familiarity, highlighted sections of text and coded this under initial descriptive titles. (3) Preliminary themes were constructed from codes by Author 1 using the topics asked in the interview questions as a guide for determining what interview content should be analyzed. These topics comprised rationale for decision to participate, benefits, areas for improvement, expectations, experiences of being in the group, experiences of singing, and learnings. (4) Authors 2 and 3 independently reviewed this preliminary construction of themes from coded extracts and either agreed with the assignment of codes or offered alternative perspectives. (5) An iterative process ensued whereby Author 1 recoded data based on feedback and further consultation with Authors 2 and 3 until consensus suggested that the themes and codes captured participant experiences related to the topics of interest. Saturation was achieved when no new codes or themes were emerging from the data. (6) Authors 1 and 2 met to further refine the themes and codes. This process involved the rewording of some theme descriptions and collapsing of others. (7) Author 3 reviewed this final iteration and suggested a few minor amendments. (8) Authors 1, 2, and 3 independently reviewed the coded extracts to ensure that participants’ experiences were authentically captured in the themes. (9) Relationships between themes were then examined with reference to previous literature to construct a framework explaining how participants in this study experienced TGS.

## Results

Twelve PwD/FCG dyads were recruited for the project and of those, nine PwD (five women, four men, mean age = 79.1, range = 57–89, *SD* = 9.5; mean MMSE score = 19.1, range = 10–26, *SD* = 4.8) and their FCG (five women, four men, mean age = 75.7, range = 61–90, *SD* = 10) completed 20 TGS sessions and participated in the interviews (75% completion rate). Eight dyads were in a spousal/partner relationship and one PwD was being cared for by her daughter. Eight dyads were born in Australia and spoke English as their first language, and one dyad had immigrated from the Ukraine in the 1960s and spoke English as a second language. Musical history was mixed with three PwD/FCG dyads who both had choral/singing experience, four dyads where one partner (PwD = 3, FCG = 1) had singing or choral experience, and two couples who had never sung in choirs before. Three dyads withdrew before completing 20 sessions. One FCG whose family member (PwD) died during the project was encouraged to continue but chose to withdraw. Another dyad completed the mid assessment but did not attend the full 20 sessions or post-assessment despite several follow up phone calls. A third dyad withdrew after the first session citing ill health as the reason for withdrawal.

An initial iteration with four themes (26 codes) developed by Author 1 was presented to Authors 2 and 3 who suggested reconceptualization and the re-organization of codes. Author 1 incorporated these suggestions and further iterations were developed involving input from all three authors over several weeks. As Authors 1 and 2 facilitated the TGS, observations during sessions may have led to pre-assumptions that the experience was positive for participants. To mitigate this risk, Authors 1 and 2 were mindful of these pre-assumptions and made efforts to minimize any influences during the data analysis. Further, Author 3 was not involved in the TGS sessions, had no relationship with participants, and was therefore able analyze the data from a more impartial perspective. Saturation was reached when new themes ceased to emerge from the data and all three authors agreed that codes were appropriately categorized within each theme. Final consensus between the three authors led to the emergence of five themes (17 codes) from the qualitative interview data: Theme (1) the TGS intervention included supportive therapeutic and design features that enhanced participant experience; (2) TGS made singing more accessible for PwD and their FCG; (3) TGS fostered new empathetic friendships for PwD and FCG; (4) TGS supported relationships between PwD and FCG; (5) PwD and FCG experienced enhanced personal wellbeing as a result of TGS. **Table 2** includes a summary of themes and corresponding codes.

**Table 2:** Themes and codes explaining participants’ experiences of therapeutic group singing (TGS)^∗^.

Theme and definition	Codes and definitions
*Theme 1. Therapeutic facilitation and design.* The intervention included supportive therapeutic features that enhanced participants’ experiences (*n* = 2 codes)	*Structure.* Several practical aspects of the TGS sessions supported participation
	*Facilitators.* The organization, engagement and enthusiasm demonstrated by the facilitators was appreciated
*Theme 2. Accessibility.* TGS made singing more accessible for PwD and their FCG (*n* = 5 codes)	*Encouraging and accepting.* PwD felt supported and this made them feel comfortable in the TGS
	*Continuing singing.* Some participants found that TGS meant they could continue with their lifelong passion for singing
	*New experiences.* Some participants who had never sung in a choir before found that TGS ignited a new passion
	*Singing is valuable.* Singing was thought to have a number of benefits for PwD, and FCG were also surprised to find that there were benefits for them as well
	*Sustainability.* There was concern about the ongoing accessibility to the singing group following cessation of research funding
*Theme 3. Empathic friendship.* TGS fostered new supportive friendships for PwD and FCG (*n* = 3 codes)	*Affinity.* Shared experiences helped participants to bond as a group
	*Empathy.* FCG valued the supportive new friendships that they developed with others who understood
	*Ripple effects.* Opportunities for connection outside TGS sessions were also sought and valued
*Theme 4. PwD/FCG Relationship.* TGS supported relationships between PwD and FCG (*n* = 2 codes)	*Togetherness.* For some dyads, singing had been central to their relationship for many years and they enjoyed being able to continue together.
	*Mutual benefits.* Other dyads experienced new and unexpected benefits from participating in singing together
*Theme 5. Personal wellbeing.* TGS led to positive individual experiences for PwD and FCG (*n* = 5 codes)	*Confidence.* TGS boosted social confidence and aroused feelings of pride
	*Self-identity.* TGS led to experiences of reconnection with self-identity and purpose.
	*Mental stimulation*. TGS promoted learning, memory and skill development
	*Enjoyment.* TGS boosted mood and feelings of happiness
	*Evokes memories.* TGS evoked discussion about meaningful memories


*^∗^Codes are not mutually exclusive.*

### Theme 1: *Therapeutic Facilitation and Design*. The Intervention Included Supportive Therapeutic Features That Enhanced Participants’ Experiences

#### Structure

Participants appreciated practical aspects of the sessions including the use of familiar participant-selected songs, opportunities to learn new and more technical pieces of music (for example, part singing), diverse instrumental support (guitars, keyboards, banjos, percussion), power point displays for lyrics, afternoon tea following singing, and regular email communication between sessions. One participant thought that the “size of the group” (FCG10) with 18 core members worked well. Participants further explained how they enjoyed opportunities to perform:

We sing the sort of songs that I like to sing (PwD1).
We’re learning lots of things… Singing in different ways aren’t we. We’re learning to use the instruments and that’s something new isn’t it (FCG12).
I’m glad they’ve got the things up on the boards because…I get my words wrong a lot of the time (PwD8).
Getting together afterwards… everyone does appreciate that catching up on how everyone’s week’s been (FCG12).
Yes, and it’s the size of the group as well. You can get around everybody. Whereas if it was any bigger, I think that might cause some problems. We perhaps wouldn’t be as close to them as we are with these people. And if anyone can’t come, there’s always enough so it’s not too small either (FCG10).
And to be able to perform a couple of times, that’s been great hasn’t it. We enjoy doing that too with everybody else (FCG2).

#### Facilitators

There were a number of comments suggesting that participants valued support provided by the two facilitators. For example, participants commented on facilitator enthusiasm, organization, regular information emails, and take-home practice tracks that supported singing between sessions and could be shared with extended family members and friends. One participant also appreciated the involvement of students during part of the program:

You guys have been excellent organizing it… you’re a key part of it. Your enthusiasm is catchy (FCG4).
And I do like having the students as part of it because I think they bring something else to the group as well… You can see that they develop as well and that’s rather nice to see as well that they gain in confidence. The longer they’re with the group as well – so it’s good to be, that you’re part of their learning as well. That it’s not just for us – that it has another purpose – a little bit as well (FCG12).

### Theme 2: *Accessibility*. Therapeutic Singing Group Made Singing More Accessible for PwD and Their FCG

#### Encouraging and Accepting

This specialized therapeutic singing group for PwD and FCG created a supportive environment where participants felt comfortable. For PwD, this meant they could participate in singing without being restricted and were able to experience a sense of belonging and affinity with others. FCG appreciated that this leveling environment was free from judgment despite any difficulties that participants might be experiencing:

I was allowed to sing in the manner that I wanted to sing and not restricted (PwD1).
It’s just a very comfortable feeling. Nobody is judging anybody else (FCG2).
The whole objective of this group is to recognize people with their difficulties rather than just being a bland choir where everyone is expected to be at the same level. It’s very supportive (FCG8).

#### Continuing Singing

Some participants had been involved in choirs and musical groups for most of their adult lives. However, symptoms of dementia meant that traditional community choirs and music groups were now too cognitively and socially demanding, and they had stopped attending. For these participants, therefore, an environment of acceptance created in this group meant they could continue to access and engage in their lifelong passion for singing:

You (referring to PwD12) were finding it more and more difficult to follow a score (FCG12). Yes, yes (PwD12)… So that transition has been really beneficial because it was a time of sadness for you not being able to continue with the (name of another choir). So, from that point of view it’s been really beneficial to have something to replace that – that’s still challenging (FCG12).

#### New Experiences

Other participants had never actively engaged in singing or music before, and accessibility to a therapeutic singing group ignited a new passion.

We’ve got people in this group who have never sung before. And yet, at that first concert we did, I know that [another participant’s name] really wanted to do it and she loved it… afterwards – she was just so happy. She said – I’ve never done anything like this before. You see people who have never done it before are discovering music and are getting this wonderful benefit too – it’s amazing (FCG2).

#### Singing Is Valuable

There was a general recognition that accessibly to group singing is valuable regardless of whether participants had previous music/singing experience or not. For some participants, this belief derived from expert opinions. Others who had been involved in singing before felt that benefits for PwD and FCG from the group were greater than they had previously realized:

There is some correlation suggesting that singing for people with Alzheimer’s is a good thing. Dr [name] suggested that this might be good for PwD1, so that’s why I was keen for PwD1 to have a go (FCG1).
I have always had music. And when mum came to stay with us, we still did the same things. But after joining this choir, we started to realise that it was more important. More beneficial. The benefits that have come out of joining the choir have made us aware of just what music can mean to people and different situations. It’s also made us probably have more music in our lives (FCG2).

#### Sustainability

However, this recognition of the value of group singing for PwD and their FCG also led to concerns about the ongoing accessibility following cessation of research funding:

Everybody’s talking about when’s it going to finish. And they’re not just talking about it – they’re really concerned about it. I know it’s research – and I know it’s incredibly important and I think it’s wonderful that its happening, but I think it’s such a shame that when the people are in the here and now, that they’re actually benefitting from it. It’s like being given a trial drug and then it fixes you but you can’t keep going (FCG2).
I would like that the group goes for ever and ever. That would be very nice (FCG9).

### Theme 3: *Empathic Friendship*. Therapeutic Group Singing Fostered New Supportive Friendships for PwD and FCG

#### Affinity

Singing with other people who had an “affinity” (PwD2) with similar life experiences, challenges and musical interests resulted in “special bonds” (FCG12) between participants:

We’re drawn together for a common reason… there’s just that feeling I think of… (FCG2) … belonging (PwD2), familiarity, friendship… (FCG2).
We have some affinity… I feel like I’m in the right place. Well – you’re surrounded by music and we’re all singing the same thing. And we’re listening to the same thing. Interested in the same thing. We’re all sort of a beautiful combination (PwD2).
I think that’s gone beyond the music, which is fantastic and you go away feeling good because you’ve been singing but then you’ve also met all these people that you can just sit and talk to. And I think that’s an unbelievable benefit…, and the longer the group is together, then the more that really works I think. That’s really important because we form special bonds (FCG12).
I think mainly… well we benefited greatly from the friendships…The first time we went… we sort of clicked with the people that are in the group and we both enjoyed singing (PwD10).

#### Empathy

FCG valued the supportive new friendships that they developed with other caregivers. This expression of empathy among caregivers was particularly recognized during critical times such as illness or during bereavement after “losing a partner to dementia” (FCG2).

Meeting the other carers has been really good. And sometimes you don’t have to say anything, but then at other times, you can. So that’s really been lovely because you don’t find that anywhere else – well I haven’t. It’s pretty isolating and a lot of friends and other people don’t understand… Even my siblings don’t understand the way they do (FCG2).
I think it’s quite tremendous how that group has really bonded as a group. And that was brought on so very strongly for me when PwD12 was sick for that month – how everyone was just so supportive of me, which I found was absolutely fantastic. That was very beneficial for me (FCG12).
And then there’s [name of volunteer bereaved after losing her husband to dementia]. I think she finds the group helpful too. It’s the camaraderie… the other carers being there – that’s so special. They know – they understand… And so that support adds another dimension that comes from the choir… And in our situation, that situation will grow (FCG2).

#### Ripple Effects

Participants also valued opportunities to connect and meet up with one another outside the singing sessions. These connections had ripple effects whereby participants made other social connections beyond the singing group:

I was going to suggest to a get-together during the holidays – it’s going to be a long time… Because it would be too long to wait until we get back to see each other again (FCG9).
And to be able to perform a couple of times, that’s been great hasn’t it. We enjoy doing that too with everybody else (FCG12).
PwD1 – he’s a member of [social organisation] and they haven’t seen him there for years but he turned up last Monday night and he sat with [my husband] the whole night… I said to [my husband] he obviously feels safer and he knows that you know and he trusts you (FCG2).

### Theme 4: *PwD/FCG Relationship*. Therapeutic Group Singing Supported Relationships Between PwD and FCG

#### Togetherness

PwD/FCG dyads explained how being able to participate in the singing group together was important to them. For some, singing together had been “central” (FCG12) to their relationship, and they valued the opportunity to continue being part of a singing group that was supportive of people with dementia.

Being able to go to choir together has been something that we’ve done for so long. So, to be able to keep up a musical interest together – that’s something we’ve really enjoyed doing for always – something that’s pretty central to our relationship. So, to be able to continue in doing that is really valuable to us … I think it’s really key that we’ve been able to do this (FCG12).
I was excited when I learnt that I could also be involved… It wasn’t just – did my mum want to – but do you both want to… it was something that we could do together that was a happy thing (FCG2).

#### Mutual Benefits

Several FCG participants had initially joined to support their loved one with dementia, but found that there were benefits for them as well. One couple spoke of past challenges in their relationship where FCG8 had sung in choirs throughout their marriage, and although PwD8 had also wanted to sing, this had not been possible before:

In the beginning – I thought it would be all for PwD10, but it’s for me as well. And um I quite enjoy it. And our family are happy because we’re enjoying it and that (FCG10).
I’ve always loved choral work and participated in it, and I thought – well this is the time for the two of us to participate in singing together… I love it – but it was always the things I had done. It’s a double benefit – I get my own self enjoyment from it and I’m conscious that the work you are doing is invaluable to PwD8 (FCG8).

### Theme 5: *Personal Wellbeing*. Therapeutic Group Singing Led to Positive Individual Experiences for PwD and FCG

#### Confidence

Participants said that singing with the group boosted their social confidence and aroused feelings of pride:

I think we’ve got more confidence to meet up with new people – and we can get up and sing and think nothing of being nervous. Gives you lot of confidence (PwD10).
How does being part of a singing group make you feel (Interviewer)? Good (PwD11 and FCG11 together). Yes, it really does. Proud yes! …Oh yes – I’m very proud. Hmm (PwD11). Oh yes (FCG11).

#### Self-Identity

Some PwD participants also felt that being a member of the singing group had provided them with a sense of purpose and reconnection with their past and self-identity. PwD8 explained how despite having word finding difficulties, she was able to reconnect with her past as a counselor:

It’s something to live for. I was still a little bit less than I am now – in being able to find the words and things – and the first day we went, [other participant] they were anxious. You could tell, and somehow or other I was just able to talk to one of them. I was really thrilled, because that was me (PwD8).

FCG4 explained how singing was part of his partner’s identity and expression of wellbeing:

*“If there is singing, then life is probably ok, but when there isn’t any singing, then life is not ok” (FCG4)*.

#### Mental Stimulation

Participants felt that music and singing were mentally stimulating and promoted learning and memory among PwD. FCGs explained how there was an association between having singing on a Friday and remembering the day of the week, and that PwD participants had learnt and remembered new songs:

He generally remembers when he realises it’s Friday – he knows he’s got singing, so that’s a good thing (FCG1). I don’t remember Thursday but Friday (PwD1). No, he doesn’t remember what he’s doing the other days of the week, so yeah, certainly he puts Friday and singing together (PwD1).
You see PwD4 can’t read the words – most of the songs – she knows off by heart, but she seems to know a few new ones now too (FCG4).

#### Enjoyment

Group singing boosted mood and led to feelings of enjoyment and happiness:

I think it always does the heart good to ah sing. I think it raises the endorphins and it always does – yes – it’s a very good thing to do. Singing is a good thing to do. And we are having fun – it is a lot of fun… You can completely switch off from everything else. It’s a very special time of the week (FCG12).
I never was participating in singing before, and I’m very happy that we did. Extremely happy that we did. For PwD9 it only works for a short time, but for me it works really for the whole week – I just keep thinking when it’s next and happiness comes to it (FCG9).

#### Evokes Memories

Singing and music were associated with meaningful memories from the past. Several participants reminisced about their younger days and how music and singing were connected with family, social gatherings, and past performances:

The family have always been keen on music. Even when we were going to school, we’d play tennis of a Saturday afternoon and we’d finish up around the piano. We’d been used to that – well I have – all my life (PwD10).
We used to have a lot of parties at our place (laughs) yes and ah – you know – mum playing the piano and singing – you know (PwD8).

## Discussion

This pilot study aimed to explore how community-dwelling PwD and their FCG experienced therapeutic group singing over 20 weeks. Qualitative data from semi-structured interviews were sought to identify participant expectations, perceived benefits (if any), experiences of the therapeutic singing group, and recommendations for facilitation. In addition, we planned to draw on the presented qualitative analysis (alongside statistical analyses from the parent feasibility study reported elsewhere) to inform the planning of a larger randomized controlled trial. This study also adds to a small but growing body of research exploring the influences and effects of community group singing for PwD/FCG dyads.

**FIGURE 1:**
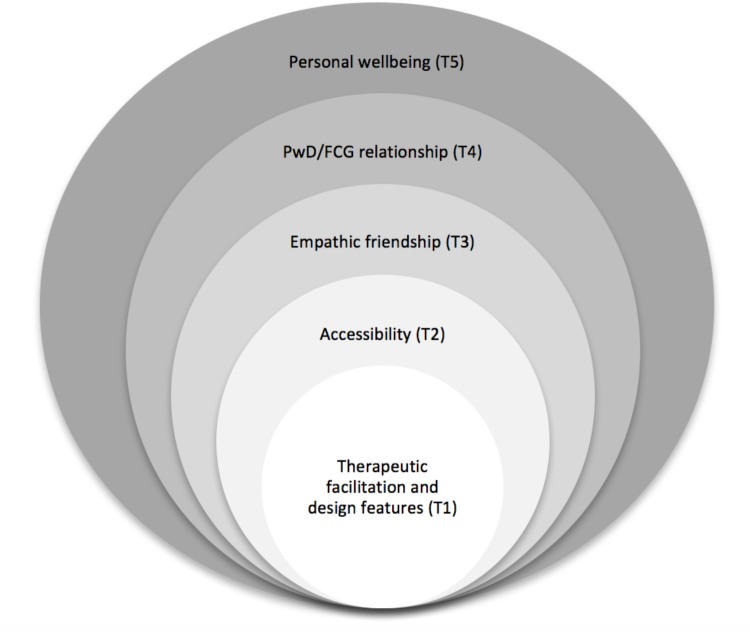
Conceptualization of therapeutic group singing for community-dwelling people with dementia and their family caregivers.

The experiences of participants in our therapeutic singing group were extremely positive and suggest that there is a need for meaningful, focused activities in the community, such as group singing, that community-dwelling PwD and their FCG can attend together. We have developed a model explaining relationships between themes emerging from this study. This model suggests that therapeutic facilitation by credentialed music therapists, with design features tailored for PwD and FCG dyads, provided supportive structure (Theme 1), which when coupled with affinity between group members and the fact that PwD and FCG could attend together, mediated improved accessibility to group singing (Theme 2), the formation of empathic friendships (Theme 3), benefits within PwD/FCG relationships (Theme 4) and wellbeing for both as individuals (Theme 5; **Figure 1**). These findings align with Kitwood’s model of person-centered care, which recognizes 12 concepts of care as central to supporting personhood and wellbeing for PwD (Kitwood, 1997). In this project, TGS facilitation underpinned by these concepts of person-centered care not only led to positive experiences for PwD, but also the development of supportive relationships and wellbeing for FCG.

Our findings support previous research explaining multiple benefits from singing groups for community-dwelling PwD/FCG dyads (Camic et al., 2013; Davidson and Almeida, 2014; Osman et al., 2016). There are some parallels with Unadkat et al.’s (2017) “group singing model in dementia for couple dyads” which evolved from an examination of community-dwelling spousal couples’ experiences across various singing groups, and McDermott et al.’s (2014) “psychosocial model of music in dementia” which emerged from thematic analysis of interviews with PwD and their families, care home staff, and credentialed music therapists. Our research builds on these conceptual understandings with an examination of group singing facilitated by credentialed music therapists for community-dwelling PwD/FCG in various relationships, including spousal and parent/child.

Participants in this project spoke of their appreciation of the facilitators’ attention to their therapeutic needs (Theme 1). As described previously, the TGS intervention, facilitated by two credentialed music therapists, was informed by Kitwood’s theory of personhood, which recognizes the capacity for wellbeing and retained strengths among PwD when environmental conditions include humanizing opportunities for meaningful and respectful social interaction and personal growth (Kitwood and Bredin, 1992; Kitwood, 1997). In keeping with Kitwood’s 12 concepts of person-centered care, sessions included spontaneous modulation of musically based elements (timalation) where participants were encouraged to contribute skills and ideas (recognition, play, creation), direct the content of sessions (negotiation, collaboration), and over time as they gained confidence (validation, holding), take increasing ownership of the singing group (negotiation, facilitation) (Kitwood, 1997). We also ensured that practical aspects of the sessions supported experiences of success. For example, power-point slides were used to display song lyrics rather than song books as they made it easier to read lyric lines and find songs while also increasing eye contact and social engagement. Access to supportive environments where skills and capacities are emphasized (as included in our TGS intervention) have been suggested to reduce the rate of decline in PwD (Kitwood, 1997).

The importance of effective therapeutic singing group facilitation for PwD/FCG dyads identified in our research, was also recognized by Unadkat et al. (2017) in their study examining various community singing groups, and McDermott et al. (2013, 2014) who explored group singing facilitated by credentialed music therapists. While Unadkat et al. (2017) did not identify the qualifications of facilitators in their study, they did capture PwD/FCG dyads’ positive and negative experiences and concluded that effective facilitation encourages belonging, equal participation and social inclusiveness among group members. Our analysis of qualitative interviews further suggests that PwD/FCG dyads experience these psychosocial benefits when TGS is facilitated by credentialed music therapists. Therefore, consistent with recommendations made by McDermott et al. (2013, 2014), we believe that credentialed music therapists with both therapeutic qualifications and music expertise provide optimal social, emotional and physical conditions for PwD and FCG attending singing groups. Indeed, there is considerable evidence demonstrating a need for expert therapeutic leadership for groups involving PwD and FCG owing to the high incidence of complex mental health issues such as depression and anxiety (Prince et al., 2016).

Therapeutic facilitation in the current research intervention created an accepting and supportive environment that improved accessibility to group singing (Theme 2). This notion of accessibility was described as an environment within the group where participants felt comfortable about attending without fear of “judgment” (FCG2) or being “restricted” (PwD1). Acceptance was largely impacted by the “affinity” (PwD2) that participants felt with one another. It is possible that a normalizing environment with others who had similar challenges provided respite from commonly reported experiences of stigma among PwD (Batsch and Mittelman, 2012), as described by participants in this study who felt ostracized from general choirs and had stopped attending.

Singing, combined with the affinity among group members led to the development of supportive friendships among participants (Theme 3). In particular, the bonds developed from group singing in this study were valued by the FCG participants. Strains on FCG including conflict and lack of awareness from other family members and social isolation increase their risk of mental health conditions such as depression and anxiety (Greenblat, 2012). FCG participants in our study spoke of these strains and stated that the empathy they experienced from and for other FCG was something they “didn’t find anywhere else” (FCG2) even among other family members. Group singing, which is known to have a strong impact on the development of social connections among people with chronic health conditions (including dementia) and also healthy populations (Gridley et al., 2011; Clark and Harding, 2012; McDermott et al., 2013; Reagon et al., 2016), acted as a conduit for the development of important new relationships among FCG. Participants in this study further suggested that the importance of these bonds between FCG would likely grow with decline in health and eventual death of their loved ones with dementia. In support of this observation, our therapeutic singing group attracted two volunteers who were both recently bereaved following the death of their loved one with dementia.

PwD/FCG dyads explained how attending the singing group together supported their relationship (Theme 4). Our intervention design that involved both PwD and FCG in the singing group together was informed by recommendations suggesting that interventions promoting togetherness, shared meaningful experiences, and enjoyment in the here and now improve relationship quality (Wadham et al., 2016). Consistent with other research modeled on these recommendations (Camic et al., 2013; Davidson et al., 2014; Osman et al., 2016; Unadkat et al., 2017), our findings suggested that dyads appreciated being able to attend the singing group together. For some dyads, the singing group allowed them to continue their musical interest together, while others enjoyed learning and participating in singing together for the first time. Further, we were surprised to hear from participants that opportunities for involvement in meaningful activities that they both enjoyed doing together were not readily available in the community, and they were very concerned that the singing group would discontinue with the cessation of research funding. Fortunately, we were able to source further funding and the group was able to continue beyond the research period. Based on our findings and other research (Osman et al., 2016), it would appear that there is a demand for greater accessibility to sustainable singing groups where both PwD and FCG can attend together.

Individual benefits from group singing reported by participants in this study (Theme 5) are widely reported. Evidence from systematic reviews recognize the positive influence from active group singing participation on outcomes measuring quality of life, mood, anxiety and depression both for people with chronic health conditions including dementia (Clark and Harding, 2012; McDermott et al., 2013; Reagon et al., 2016) and healthy populations (Clift et al., 2008). Consistent with our findings, other qualitative research examining group singing for PwD/FCG dyads also suggest that participants experience mental stimulation, enjoyment, and improved feelings of social confidence and self-esteem (Camic et al., 2013; Osman et al., 2016; Unadkat et al., 2017). In addition, a number of participants expected singing to be particularly valuable for PwD based on media reports and comments they had heard from experts (for example, their medical doctor) suggesting that singing is broadly recognized as beneficial by society. A number of FCG in this study also commented on the way PwD were able to learn of new songs and retain memory for these songs from week to week. While this phenomenon is controversial, it has been reported by participants in other singing groups for PwD and FCG (Camic et al., 2013), and in a detailed case study demonstrating immediate and delayed recall of an unfamiliar song by a 91-year-old woman with advanced dementia and no previous musical training (Baird et al., 2017). Further, recent research involving original group songwriting for PwD has demonstrated an ability to recognize and build on previously unknown musical material from one week to the next (Baker and Stretton-Smith, 2017). This capacity to learn new songs is fascinating to observe and deserves further research.

### Limitations

The current study only investigated experiences following a 20-week intervention. This relatively short intervention period could be considered a limitation owing to the evolving and dynamic nature of the singing group with participants commencing at different times coupled with the degenerative nature of dementia. Nonetheless, in examining the first 20 weeks of the singing group, we also captured the group through its developmental stages with the formation of new relationships and personal changes such as increased confidence. Since this therapeutic singing group included spousal (*n* = 8) and parent/offspring (*n* = 1) dyads, we are unable to differentiate how the therapeutic singing group might be experienced differently across various caregiver–recipient relationships. It is also worth noting that we interviewed PwD and FCG participants from each dyad together, and while we feel that our PwD participants made significant contributions to the data (in spite of their dementia), it is possible that FCG participants are over-represented. Finally, the two interviewers (Authors 1 and 2) also facilitated the TGS sessions and it is possible that this relationship with participants influenced their responses and researcher pre-assumptions may have influenced the analysis of data. To deal with this, the third author (who was not involved in interviews or group facilitation) carefully read the data and assisted with the analysis process.

### Recommendations

Based on the findings from this project and others, therapeutic group singing appears to make a positive short-term difference to the lives of community-dwelling PwD and FCG (Camic et al., 2013; Osman et al., 2016; Unadkat et al., 2017). However, systematic reviews suggest that knowledge about longer-term influences of TGS for community-dwelling PwD/FCG dyads is limited (Clift et al., 2008; Clark and Harding, 2012; McDermott et al., 2013; Reagon et al., 2016). Attendance in singing groups is a lifestyle choice, and it is also possible that there may be cumulative benefits over time such as delayed disease progression in symptoms dementia, improved coping among FCG, and reduced experiences of social isolation for both PwD and FCG leading to significant impact on long-term quality of life. Therefore, it would be interesting to explore ongoing health and well-being outcomes as well as experiences of TGS participants over the longer-term. Further, dementia is a degenerative disease and so an exploration of PwD’s engagement in regular group singing from early through to later stages of the disease trajectory is relevant. Given the relationship between early interventions that improve social engagement, health and emotional wellbeing for both PwD and FCG and delayed admission to residential care for PwD (Greenblat, 2012; Prince et al., 2015; Farina et al., 2017; Frankish and Horton, 2017), it would also be worthwhile embedding a health economics health evaluation into a longer-term study.

Consistent with previous research, our findings suggest that facilitator expertise may impact the effectiveness of group singing for community-dwelling PwD and FCG (Unadkat et al., 2017). Therefore, it would be interesting to examine and compare various facilitation models. Such models might include singing groups for PwD/FCG dyads facilitated by: (1) credentialed music therapists; (2) community musicians; (3) credentialed music therapists and community musicians working together; and (4) community musicians overseen and trained by credentialed music therapists. It may be that different models would be suitable depending on the degree of recommended therapeutic input. For example, if participants were experiencing high levels of depression and anxiety, then a credentialed music therapist with expert therapeutic and music skills might be recommended. Alternatively, a professional musician may be more suitable for leisure singing groups where members have good mental health and an interest in musical skill development and performance opportunities. Finally, given low available numbers of credentialed music therapists compared with growing numbers of PwD, a combination model involving professional musicians who receive training and guidance from credentialed music therapists might best meet a potential high demand from PwD/FCG dyads for community group singing (Osman et al., 2016).

Participants in this study spoke about how they enjoyed giving performances. Performances by PwD/FCG singing groups demonstrate capacity and success, even among people with advanced dementia (Baird and Samson, 2009), and may contribute to a greater understanding of dementia among members of the public. A greater understanding and awareness of dementia among the general public has the potential to reduce negative social perceptions and stigma leading to improved empathy (Batsch and Mittelman, 2012). Therefore, it would be interesting to examine audience and social media responses to public performances by PwD/FCG singing groups.

## Conclusion

Participants in the current project were extremely positive about their experiences of this therapeutic singing group. This enthusiasm was evident from participants who had previous musical and singing experience and also by those who had never sung in a group before. A therapeutic singing group attended by PwD/FCG dyads together provided a supportive environment where participants had an affinity with one another. Within this environment PwD and FCG participants developed supportive new friendships and experienced many personal benefits, including feelings of success, improved confidence, enjoyment, and mental stimulation. For PwD participants, the singing group was a place where they could demonstrate strengths and skills rather than deficits. FCG participants particularly valued the empathy and understanding they shared with other caregivers. These psychoemotional, social and cognitive benefits are thought to promote self-identity, healthy relationships, and wellbeing for both PwD and their FCG (Greenblat, 2012). Therapeutic singing groups for community-dwelling PwD/FCG dyads are not readily available in Australia, where this research was conducted, despite international evidence demonstrating a growing demand for such services (Osman et al., 2016). Therefore, further exploration of various models facilitated by credentialed music therapists and/or professional musicians might increase the availability of sustainable therapeutic singing groups leading to improved quality of life for community-dwelling PwD and their FCG.

## Author Contributions

IC and JT were responsible for the design, intervention implementation, qualitative interviews, and qualitative data collection for this study. IC, JT, and FB contributed to the qualitative data analysis and writing of the manuscript.

## Conflict of Interest Statement

The authors declare that the research was conducted in the absence of any commercial or financial relationships that could be construed as a potential conflict of interest.
